# An application of statistics to comparative metagenomics

**DOI:** 10.1186/1471-2105-7-162

**Published:** 2006-03-20

**Authors:** Beltran Rodriguez-Brito, Forest Rohwer, Robert A Edwards

**Affiliations:** 1 Computational Science Research Center, San Diego State University, San Diego, USA; 2 Center for Microbial Sciences, San Diego State University, San Diego, USA; 3 Department of Biology, San Diego State University, San Diego, USA; 4 Fellowship for Interpretation of Genomes, Burr Ridge, USA

## Abstract

**Background:**

Metagenomics, sequence analyses of genomic DNA isolated directly from the environments, can be used to identify organisms and model community dynamics of a particular ecosystem. Metagenomics also has the potential to identify significantly different metabolic potential in different environments.

**Results:**

Here we use a statistical method to compare curated subsystems, to predict the physiology, metabolism, and ecology from metagenomes. This approach can be used to identify those subsystems that are significantly different between metagenome sequences. Subsystems that were overrepresented in the Sargasso Sea and Acid Mine Drainage metagenome when compared to non-redundant databases were identified.

**Conclusion:**

The methodology described herein applies statistics to the comparisons of metabolic potential in metagenomes. This analysis reveals those subsystems that are more, or less, represented in the different environments that are compared. These differences in metabolic potential lead to several testable hypotheses about physiology and metabolism of microbes from these ecosystems.

## Background

Metagenomics describes the functional and sequence-based analysis of DNA isolated from environmental sample without first culturing the associated microbes [1]. Four viral and four prokaryotic shotgun metagenome datasets have been published [2-8]. The acid mine drainage (AMD) metagenome data set was taken from a low complexity environment and includes slightly more than 10 Mb of sequence in 2,455 contiguous sequences (contigs) and ~8,000 predicted protein sequences [8]. The Sargasso Sea metagenome data set is from a more complex environment and includes 788 Mb of sequences in 809,112 contigs, and approximately a million predicted protein sequences [7].

Another conceptual "metagenomic" library can be constructed from the combined sequence data collective generated over the last 30-plus years of DNA and protein sequencing and deposited in the international databases. In contrast to the environmental metagenome libraries, the collective metagenome was built by the incremental addition of sequences from many different sources. The SEED database, developed by the Fellowship for Interpretation of Genomes (FIG), is an annotated non-redundant database, compiled from several sources including GenBank (including GenBank's non-redundant and refseq databases), Swiss-Prot, KEGG, and from genome sequencing centers. At the time of analysis, the SEED database contained 1,731,649 proteins (not including the environmental samples; Table 1). Although the sampling of sequences that constitute this library is not random, the SEED essentially represents essentially all known genomic complexity.

FIG pioneered the use of subsystems to annotate both complete and partial genome sequences [9]. Subsystems are biochemical pathway, fragments of pathways, clusters of genes that function together, or any group of genes that any annotator considers to be related. The subsystems are annotated across genomes by the annotators, providing the most reliable and consistent annotations within and between genomes. The subsystems-based annotations are ongoing and at a given point in time the subsystems represent the snapshot of the best available annotation of the SEED database.

Comparing metagenome samples could lead to the identification of signature functions associated with each metagenome sample, however this analysis requires reliable statistical techniques that are not only robust but are rapid to perform with hundreds of thousands or millions of data points per sample.

Here the Sargasso Sea and AMD metagenomes were compared with the SEED database to identify statistically significant differences in subsystems composition. We hypothesized that there were few barriers to the transfer of subsystems between environments and therefore certain subsystems were enriched by selection in those environments where they were important. We used a difference of medians analysis to identify those subsystems that have a statistically significantly presence in each of the metagenomes. These analyses provide a framework for the statistical comparison of metagenomes.

## Results

### Determination of statistically significantly different subsystems

A difference between medians calculation was applied to rapidly identify statistically significant differences between metagenomes. This technique has several advantages over other possible statistical methods that could have been applied. For example, the difference between medians is extremely rapid for the calculation of differences between subsystems from different samples, and the method does not depend on the distribution of samples. The source code and step-by-step description of the method are provided as part of the supplemental material [see Additional Files 1 and 2].

### Number of samples needed to identifying significant differences between metagenomes

**Figure 1:**
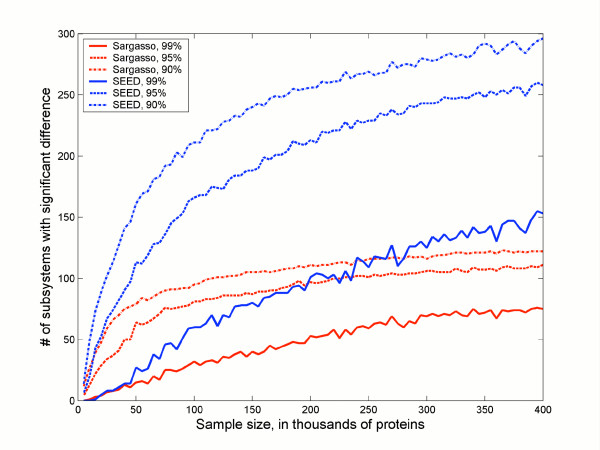
**Effect of sample size on identifying differences between phylosubsystems**. The red lines reflect the number of phylosubsystems overrepresented in the Sargasso Sea dataset. The blue lines represented the number of phylosubsystems overrepresented in the SEED dataset. Three different confidence levels (90, 95, and 99%) are plotted.

Fig. 1 plots the sample size (*S*) of proteins that were sampled against the number of significantly different subsystems when the Sargasso data was compared to the SEED data at different confidence levels. At the most stringent level (99% confidence that the difference does not occur by chance) there are about 75 unique phylosubsystems in the Sargasso sample (solid red line) and about 150 unique phylosubsystems in the SEED. In either case at least the sample size needs to approach 300,000 proteins to achieve statistical significance. In contrast, at lower confidence levels, there are more significantly different phylosubsystems, and the samples need to be smaller to detect them. Thus with a 90% confidence that the difference do not occur by error there are approximately 100 unique subsystems in the Sargasso data, and a sample size of about 150,000 proteins is required to identify them all.

The AMD dataset has many fewer phylosubsystems than the SEED dataset, and only those subsystems present in the both samples are used in the comparison to identify significantly different phylosubsystems. This limits the analysis to 284 of the 523 different phylosubsystems. Of those that are present in the AMD and the SEED, only 19 are significantly more abundant in the AMD sample (Table 2) and 58 are more abundant in the SEED dataset. Saturation was reached at ~145,000 samples.

### Subsystem differences between the SEED and Sargasso Sea metagenomes

**Figure 2:**
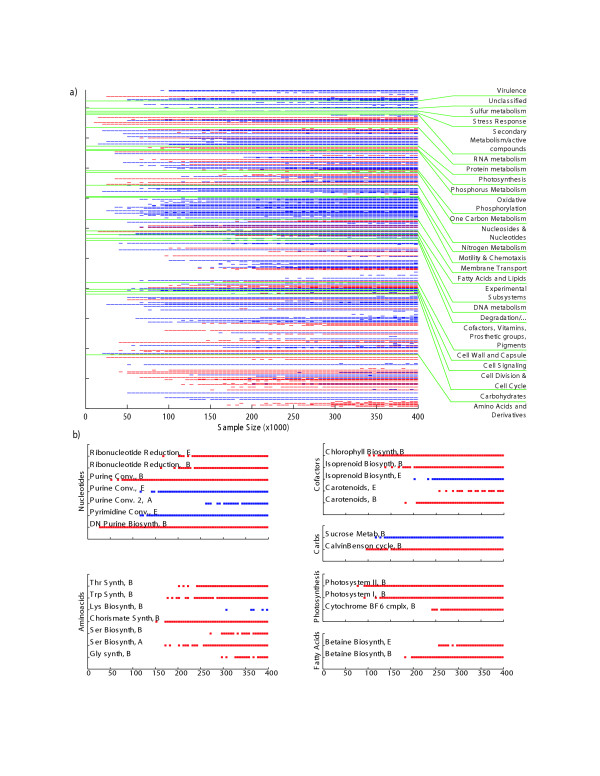
**Significantly different subsystems between Sargasso Sea and SEED datasets**. **(A). **Each subsystem was bootstrapped with between 10 and 400 samples per bootstrap, and subsystems that are significantly different with 99% and 2,000 bootstraps are highlighted. Those subsystems that are significantly more prevalent in the SEED database are colored blue, and those subsystems that are significantly more prevalent in the Sargasso Sea dataset are colored red. **(B). **Magnified view of several different subsystems. Subsystems from amino acid synthesis, carbohydrate utilization, cofactor synthesis, fatty acids, nucleotide synthesis, and photosynthesis are shown in more detail. Colors are as described for (A). E = eukaryotic subsystem, B = bacterial subsystem, A = archaeal subsystem.

Statistically different phylosubsystems between the SEED and Sargasso Sea metagenomes are shown in Fig. 2A. The effect of sample size is apparent. For example, statistically differences between RNA metabolism, oxidative phosphorylation, and membrane transporters can be detected at small sample sizes (e.g., 100,000 proteins). However, the differences between carbohydrate and amino acid metabolism, as well as most cofactors, vitamins, and pigments are not statistically different until >150,000 proteins have been sampled. Out of the 77 phylosubsystems that are significantly overrepresented in the Sargasso Sea, 69 are from the Bacteria, 5 from the Archaea, and 3 from the Eukarya.

Fig. 2B highlights some of the differences for phylosubsystems. A more detailed description of all of the subsystems with statistically significant differences in occurrence between environmental data sets is given in the supplemental material [see Additional file 3]. Some ecologically important differences between the Sargasso Sea and the SEED database are discussed below with data extracted from the Supplementary Table.

### Potential osmoregulation by amino acids in the Sargasso Sea

**Figure 3:**
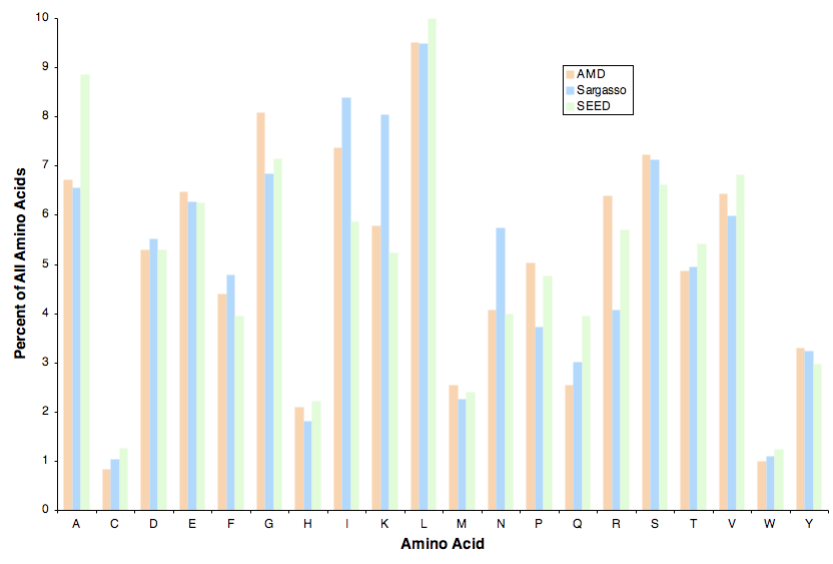
**Fraction of amino acids in metagenomes**. The fraction of each amino acid in all the predicted proteins in the three data samples was counted and compared.

The phylosubsystems involved in the synthesis of serine (S), threonine (T), and glycine (G) are overrepresented in the Sargasso Sea metagenome (Table 3). For example, there are 503 proteins per million proteins in the Sargasso Sea database that are similar to bacterial glycine synthesis proteins, and only 390 proteins per million proteins in the SEED database that are similar to bacterial glycine synthesis proteins. Exactly these three amino acids are also the most abundant amino acids found in the Sargasso Sea [10,11]. However proteins in the Sargasso Sea metagenome do not contain significantly more S, T, or G (Fig. 3), suggesting that the observed bias in amino acid synthesis subsystems and in the water samples is not related to protein synthesis. The primary organic osmolytes used by marine bacteria are small organic, uncharged, solutes that have little effect on the intracellular biochemistry, such as glycine betaine [12-15]. The subsystems for the production of betaine are also overrepresented in the Sargasso Sea compared to the SEED database (Fig. 2B). Similarly, serine and threonine are small, polar, uncharged amino acids that are perfect compatible solutes for balancing osmotic pressure. Serine has also been previously shown to be as effective as glycine betaine at protecting enzymes from the effects of increased osmolality [15]. In contrast, genes encoding the osmoprotectants proline, sucrose, and trehalose [13,14,16,17] were underrepresented in these samples. Therefore, we predict that marine microbes are synthesizing glycine, serine, threonine, and betaine as osmolytes, an hypothesis that can be tested experimentally.

### Photosynthesis in the Sargasso Sea

As previously observed [6,7], there was a strong bias towards subsystems involved in photosynthesis in the Sargasso Sea metagenome. This bias includes subsystems for the Calvin-Benson cycle, chlorophyll biosynthesis genes, the cytochrome B6-F complex, Photosystem I, Photosystem II, isoprenoid biosynthesis, and carotenoid biosynthesis.

Some phylosubsystems involved in one-carbon metabolism, including the synthesis and degradation of carbohydrates, cell walls, and capsules are more abundant in the Sargasso Sea. In contrast, the genes for the utilization of complex carbon sources including lactose, arabinose, fructose, mannose, galcitol and inositol are all underrepresented in the marine environment suggesting these are not significant sources of carbon in this environment.

### Nucleic acid and phosphate metabolism in the Sargasso Sea

Phylosubsystems involved in purine and pyrimidine *de novo *synthesis and scavenging pathways, as well as ribonucleotide reduction (scavenging ribonucleotides for DNA synthesis) are more abundant in the Sargasso Sea. Similarly, the subsystems involved in the capture of phosphate *via *the conversion of ADP to ATP coupled to oxidative phosphorylation are also overrepresented in the Sargasso sample. In contrast, nitrogen metabolism phylosubsystems are less abundant in the Sargasso than the SEED, with the sole exception of ammonia assimilation that is marginally overrepresented in the Sargasso sample at larger sample sizes. The Sargasso Sea has previously been reported to be phosphate limited. The concentration of dissolved inorganic phosphate is approximately 4 nM in the Sargasso Sea. By comparison, the North Pacific and typical bacterial minimal media have phosphate concentrations of approximately 100 nM [18,19]. Together, these results suggest that phosphate acquisition is critical for microbial growth the Sargasso Sea environment.

### Mobility of Sargasso Sea microbes

Estimates of the percentage of bacteria in the ocean that are motile vary from less than 5% to more than 80% [20,21], and there were far fewer genes encoding flagella in the marine environment compared to the SEED database. However many marine microbes are thought to use alternative, less well characterized, motility systems, such as the motility mechanism characterized in cyanobacteria [22,23] or twitching motility previously shown in marine microbes [24]. This data leads to the hypothesis that marine microbes are generally not using flagella based motility for movement, and future studies on the genomics of twitching and gliding motility may reveal hints about these mechanisms of movement.

### Subsystem differences between the SEED and AMD metagenomes

When the AMD and SEED databases were compared, only phylosubsystems that were in both the AMD and the SEED samples were included. This limited the total number of subsystems that were compared for statistically significant differences. There are far fewer phylosubsystems with significantly different distributions between the AMD and SEED datasets, and phylosubsystems that are significantly more common in the AMD dataset are shown in Table 2. The different occurrences of subsystems reflect the limited complexity of the AMD environment that contains Bacteria and Archaea [8]. The majority of subsystems that are significantly more common in the AMD data set are from archaeal proteins. In the AMD environment, the production of amino acids does not appear to be critical, and only archaeal arginine and histidine degradation and leucine and chorismate synthesis are overrepresented in these samples. Our limited selection of overrepresented subsystems in the AMD sample presumably reflects the current bias in annotated subsystems in the SEED. As the subsystems continue to evolve and expand, and the NIH Project to Annotate 1,000 Genomes [9] matures the impact of these annotations on the AMD sample and other metagenomes will highlight those areas of metabolism and physiology that are critical to survival in different environments.

### Subsystem differences between the Sargasso, SEED, farm and whale metagenomes

The SEED and Sargasso subsystems were compared to both the whale fall and farm metagenome samples [6]. For this comparison the individual whale fall samples, and individual farm samples were each combined to create two separate metagenomes. Those metagenomes were compared to the subsystems exactly as described in Methods, using the BLAST algorithm to determine similar sequences. The data shown in the supplemental material [see Additional file 4] was created using 95% confidence, a sample size of 20,000 proteins, and 20,000 replicates. This table shows each of the comparisons with the statistically significant subsystems. The normalized data was used to determine the relative abundance of each KEGG pathway in each sample [25], and these comparisons are shown in the supplemental material [see Additional File 5].

The KEGG pathways have historically focused on core metabolism, annotating enzymes that have been classified with EC numbers. In contrast, the SEED subsystems include core metabolism and the data is extended to subsystems that cover cellular processes and functions, regulation, and so forth. Although the two classification techniques are not directly comparable, and statistical confidence was not provided with the differences between KEGG pathways in the supplemental data from the previous analysis, some clear parallels can be seen between these analyses [see Additional File 5]. For example, both techniques identified that riboflavin metabolism is more prevalent in the Whale Fall metagenomes than the other samples, however according to the normalized data from Tringe *et al *folate biosynthesis is less abundant in the Sargasso metagenome than either the Whale Fall or Soil Metagenomes whereas this analysis demonstrated that there is significantly more folate biosynthesis in the Sargasso than the other samples. There were 9,311 proteins with similarity to folate biosynthesis subsystem from the SEED database in the Sargasso metagenome, 602 proteins with similarity in the farm soil metagenome and 491 proteins with similarity in the Whale Fall metagenome. In contrast, Tringe *et al*. identified 7,283 proteins, 1,253 proteins, and 889 proteins respectively. These differences are probably due to the difference in annotation of the SEED subsystems and KEGG pathways. These differences also highlight the need for continued careful annotation of genomes, and comparative analysis of different annotation systems and methods.

## Discussion

Community genome sequencing – metagenomics – can provide fine detailed analysis of the metabolism occurring in different ecosystems. However, metagenomics analysis is limited to a purely descriptive science without a rigorous statistical comparison of the prevalence of different genes in different environments. Our analysis demonstrates an application of statistics to identify those areas of metabolism that are significantly over represented in different environments.

The method described here is predicated on the expectation that genes that are more useful in an environment are more commonly found in that environment. Or put another way, there is an enrichment or selection for sets of genes in different environments. A statistical analysis, using a resampling with replacement technique, was developed to generate both the difference in occurrence of each subsystem in each sample, and to generate confidence intervals for the likelihood that these differences are observed by chance. By using these statistical techniques to compare the genetic composition of different environments, the areas of metabolism and biochemistry that are important in a particular environment, in comparison to other environments, can be identified. Like other studies, this analysis demonstrated that microbes in the surface waters of the ocean are much more likely to contain genes involved in photosynthesis than the control data set. The non-redundant database used as a control is not expected to contain large numbers of photosynthetic organisms because it is skewed towards microbial pathogens.

Our analysis also demonstrated more than 150 other subsystems that are over represented in the Sargasso Sea sample when compared to the control set. The skew in the database alone cannot explain this difference, and these subsystems must be important for survival in the ocean. Some examples, such as the synthesis of serine, threonine, and glycine, directly testable hypotheses can be generated from these analyses. For other examples, the explanation of the differences between samples may be more elusive. Several pieces of evidence will assist in determining the roles of different subsystems in different environments. For example, the inclusion of more environmental data with each sample will allude to some of the differences in metabolic potential between samples; the careful dissection of the presence of different subsystems in different organisms will identify which organism in which environment is performing the different biochemical reactions; and the extension of other techniques such as metabolic modelling into the environmental arena may provide insights into the critical biochemical mechanisms in each environment.

## Conclusion

Comparative metagenomics is a powerful mechanism for highlighting the ecological differences between environmental samples. Our analysis has generated several hypotheses that can be readily tested on microbes from the Sargasso Sea:

1. Serine, threonine, and glycine betaine are primarily being used as osmoprotectants. Increased intracellular concentrations of serine may protect and against the osmolarity of the ocean. In contrast, sucrose and trehalose are not being used as readily for osmoprotection.

2. Microbes in the Sargasso Sea are more limited for phosphate than nitrogen.

3. Microbes in the ocean are not generally using flagella based motility but are probably using one of the less-characterized mechanisms of locomotion.

4. Archaea in the AMD sample are degrading arginine and histidine.

The subsystems approach to investigating environmental genomes demonstrates the intricate interplay between the abundance of genes in the environment and the biology of that environment. In addition to answering that age-old knock-knock joke [26] by cataloging the organisms that are present in an environment and looking for novel proteins and structures, metagenomics also provides critical insights into our understanding of the physiology, biology, and ecology of an environment. Using subsystems to compare the ecology of sites that have been sampled by metagenomics can be applied to any other metagenome samples to provide similar insights into the ecology of those environments.

## Methods

### Sequence databases

The complete SEED database v4 was used as the source of all data [27]. Construction and annotation of the subsystem database is described elsewhere [9,28]. The environmental sequences were removed from the SEED database for the analyses presented here. Furthermore, any sequences with principal homology to either *Shewanella *sp. or *Burkholderia *sp. were removed from the Sargasso Sea metagenome because of contamination concerns [29]. This dataset contained 960,561 predicted proteins. The AMD data set contained 7,588 predicted proteins. For these analyses the term "protein" is used when referring to predicted proteins.

### Assignment of proteins to subsystems and phylosubsystems

Each protein from the AMD, Sargasso, or the SEED database was compared to proteins in the SEED database previously assigned to particular subsystems by the SEED annotators [9]. A protein was considered a member of a subsystem if the protein had significant similarity (designated as an E value less than 1 × 10^-20^) to another protein previously assigned to a subsystem. Each protein was also classified as Bacteria, Eukarya, and Archaea, based on the Domain assignment of the most similar protein. There were a total of 276 annotated subsystems in the SEED. Bacteria had proteins in 257 of the subsystems. Archaea and Eukarya had proteins in 132 and 134 of the subsystems, respectively. This means that each there were a total of 523 potential data points. The term phylosubsystem is used to reflect that fact that the assignments are based both on the subsystem and Domain.

### Comparisons of metagenomic databases

**Figure 4:**
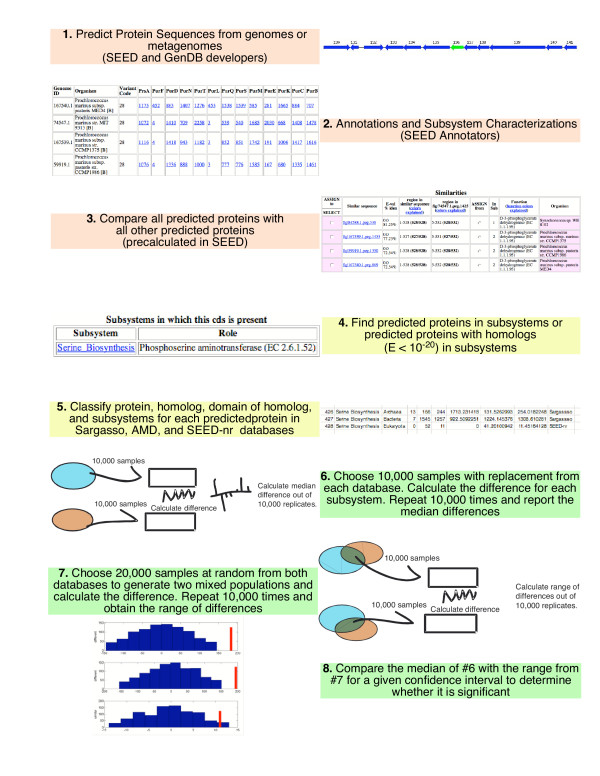
Flow chart of methods used to identify statistical differences between phylosubsystems.

A flowchart of the methods used to identify subsystems with statistically significant differences between databases is shown in Fig. 4 and described in the supplemental material [see Additional File 2]. In addition, source code to software to calculate these differences is provided as supplemental material [see Additional File 1]. In order to decide whether a subsystem was over-represented between metagenomes, a comparison between the median number of proteins assigned to specific subsystems was performed for a given confidence level. The following steps were carried out: 1) A number N of proteins were drawn at random, with replacement, from each of two metagenomes. Each protein was classified into a subsystem. Then, the difference between the two sets of samples was calculated for each subsystem, resulting on a list of differences between metagenomes by subsystems. These differences were expected to be more pronounced where the differences between metagenomes were more remarkable, but with just one set of differences this was not guaranteed. Thus, this procedure was repeated M times. Then, for each subsystem the median of all the M differences was calculated. 2) To build a confidence interval, a number N of "mixed" samples were drawn at random, with replacement, from a mixture of both metagenomes. For each sample, two random numbers were drawn, the first one to decide which metagenome would be selected, and the second one to choose a particular element of the metagenome, which was the accounted into its corresponding subsystem. This process was repeated for another set of N samples and with these two mixed sets a difference was calculated. This whole process was repeated M times again, and for each subsystem the resulting differences were ordered and used to build a confidence interval. The limits of the confidence interval for each subsystem were estimated by using the corresponding quantile elements on the ordered set of differences of each subsystem as limits to the confidence interval. For example, if M = 1,000 and the confidence level was 90%, the limits for the confidence interval were at the 5% and 96% percentiles on the ordered values of the differences for that subsystem, that is, the 50^th ^and 951^st ^elements would be the upper and lower limits on the confidence interval. 3) For any given subsystem, if the median from step 1 was inside the confidence interval determined in step 2 it was considered that the subsystems were not significantly different at the given confidence level. If the median was outside the confidence interval, it was assumed that the difference was significant at the given confidence level.

### Software to calculate the statistics

A software package that calculates the statistics from appropriately formatted files is provided as supplemental material [see Additional File 1]. The software is released under the GPL license and is available from the BMC website.

## Authors' contributions

BRB developed the statistics and compared the samples.FR contributed to the discussion on roles of subsystems in the samples. RE generated the connections between proteins and subsystems. All authors contributed to authoring and editing the manuscript.

## Supplementary Material

Additional File 1 Step-by-step description of the methods used to calculate the differences between subsystems Click here for file

Additional File 2 A tar gzip compressed archive of source code and instructions for calculating difference of medians as described here. Version 0.01 of the software, released under the terms of the Gnu General Public License (GPL). Click here for file

Additional File 3 Presence of subsystems in different environmental samples in tab-separated text format. Click here for file

Additional File 4 Presence of subsystems in the AMD, Sargasso, and SEED samples in tab-separated text format. Click here for file

Additional File 5 Abundance of selected Kegg Pathways and Subsystems that are approximately similar as detected either based on the Normalized differences of Tringe *et al *(reference 6) or using the method described herein. Note that the relative gene content of the subsystems and Kegg pathways is never identical. Click here for file

## Figures and Tables

**Table 1:** Number of genomes and protein encoding genes in the SEED database at the time of analysis. The two environmental samples are the Sargasso Sea and Acid Mine Drainage metagenomes.

***Domain***	***Number of Genomes***	***Proteins***	***Percent of all proteins***
Archaea	37	61,709	2
Bacteria	550	1,187,180	44
Eukarya	556	482,760	18
Environmental Samples	(2)	968,149	36
**Total**	**1,145**	**2,699,798**	**100**

**Table 2:** Phylosubsystems that are overrepresented in AMD dataset versus SEED dataset with 99% confidence at a sample size of 145,000 proteins.

***#***	***Subsystem***	***Classification***	***Domain***
1	Arginine degradation	Amino Acids and Derivatives	Archaea
2	Chorismate Synthesis	Amino Acids and Derivatives	Archaea
3	Histidine Degradation	Amino Acids and Derivatives	Archaea
4	Leucine Biosynthesis	Amino Acids and Derivatives	Archaea
5	Calvin-Benson cycle	Carbohydrates	Archaea
6	Embden-Meyerhof and Gluconeogenesis	Carbohydrates	Archaea
7	Methylcitrate cycle	Carbohydrates	Archaea
8	Riboflavin metabolism	Cofactors, Vitamins, Prosthetic Groups, Pigments	Archaea
9	Conserved tRNAs	Experimental Subsystems	Archaea
10	Fatty acid metabolism	Fatty Acids and Lipids	Archaea
11	Fatty acid oxidation pathway	Fatty Acids and Lipids	Archaea
*12*	*de-novo *Purine Biosynthesis	Nucleosides and Nucleotides	Archaea
*13*	*de-novo *Pyrimidine Biosynthesis	Nucleosides and Nucleotides	Archaea
14	Pyrimidine conversions	Nucleosides and Nucleotides	Archaea
15	Ribosome LSU (eukaryotic and archaeal)	Protein Metabolism	Archaea
16	Ribosome SSU (eukaryotic and archaeal)	Protein Metabolism	Archaea
17	Translation initiation factors (eukaryotic and archaeal)	Protein Metabolism	Archaea
18	tRNA aminoacylation	RNA metabolism	Bacteria
19	TTSS transporters	Virulence	Bacteria

**Table 3:** Presence of Glycine, Serine, and Threonine subsystems in the AMD, SEED, and Sargasso databases. The table is a subset of the data from the supplemental data [see Additional File 3].

**No**^**1**^	**Name**^**2**^	**K**^**3**^	**Classification**^**4**^		**# AMD**^**5**^	**# SEED**^**6**^	**# SS**^**7**^	**AMD per million**^**8**^	**SEED per million**^**9**^	**SS per million**^**10**^	**Prev.**^**11**^
206	Glycine synthesis	A	Amino Acids	Alanine, serine, and glycine	7	24	34	923	19	35	Sargasso
207	Glycine synthesis	B			2	492	483	264	390	503	Sargasso
426	Serine biosynthesis	A			13	166	244	1713	132	254	Sargasso
427	Serine biosynthesis	B			7	1545	1257	923	1224	1309	Sargasso
475	Threonine synthesis	B		K, T, M, and C	8	1070	952	1054	848	991	Sargasso

^1^Subsystem number

^2^Subsystem name as designated by the curator

^3^K: Kingdom (A: Archaea; B: Bacteria; E: Eukaryota)

^4^Classification of the subsystem. K, T, M, and C: Lysine, threonine, methionine and cysteine.

^5^Number of proteins present in the AMD sample

^6^Number of proteins present in the SEED sample

^7^Number of proteins present in the Sargasso Sea sample (SS).

^8–10^Number of proteins present in the AMD, SEED, and Sargasso samples normalized per million proteins in each sample.

^11^Statistically significant prevalence. Prev.: prevalence.
