# Supplementary material for: An application of statistics to comparative metagenomics
Source: BMC Bioinformatics. 2006 Mar 20;7:162. doi: 10.1186/1471-2105-7-162 (PMC1473205; doi:10.1186/1471-2105-7-162)
Supplement: Additional File 2 — A tar gzip compressed archive of source code and instructions for calculating difference of medians as described here. Version 0.01 of the software, released under the terms of the Gnu General Public License (GPL). [file 1471-2105-7-162-S2.PDF]

A. To count the presence of subsystems in samples

1. Start with sample 1 and select (with replacement) 10,000[a] proteins from that sample.
2. Count the number of times each subsystem is seen in sample 1.
3. Repeat with sample 2, and count the number of times each subsystem is seen in 10,000 proteins.
4. For each subsystem calculate the difference in occurrence between sample 1 and sample 2.
5. Repeat this process 20,000[a] times. Each time take 10,000 proteins from each sample and count the number of times a subsystem is seen, and calculate the difference in occurrence between sample 1 and sample 2.
6. We now have 20,000 differences in occurrence between sample 1 and sample 2 for each subsystem. Average those differences to get the median difference in occurrence between subsystems. Remember these numbers.

B. To determine the confidence intervals of the medians

1. Generate two random datasets by choosing 10,000 proteins from either sample 1 or sample 2. For each data point, both the sample and the protein within the sample are chosen at random.
2. Generate the differences between subsystems for each dataset exactly as above.
3. Repeat this random sampling and difference calculation 20,000 times.
4. For each subsystem, order the differences from least to most. Generate percentiles based on the number of samples taken. Hence, if 20,000 differences are calculated the 5th percentile will be the 1,000th difference and the 95th percentile will be the 19,001th difference. Remember these numbers.

C. To determine the statistical significance of the samples

1. For each subsystem, take the median from A6 and the 5th and 95th percentile limits from B4.
2. If the median is between the confidence limits there is no statistically significant difference between the two samples.
3. If the median lies outside the confidence limits there is a statistically significant difference between the two samples.

[a]The use of 10,000 proteins and 20,000 repeats is merely an example, and the actual numbers used will depend on the size of the dataset and the speed of the simulation.
