# Supplementary material for: An application of statistics to comparative metagenomics
Source: BMC Bioinformatics. 2006 Mar 20;7:162. doi: 10.1186/1471-2105-7-162 (PMC1473205; doi:10.1186/1471-2105-7-162)
Supplement: Additional File 5 — Abundance of selected Kegg Pathways and Subsystems that are approximately similar as detected either based on the Normalized differences of Tringe et al (reference 6) or using the method described herein. Note that the relative gene content of the subsystems and Kegg pathways is never identical. [file 1471-2105-7-162-S5.doc]

**Supplemental Table S3.** Abundance of selected Kegg Pathways and Subsystems that are approximately similar as detected either based on the Normalized differences of Tringe *et al* (reference 6) or using the method described herein. Note that the relative gene content of the subsystems and Kegg pathways is never identical.

|  |  | **Tringe *et al.* Normalized differences** | | | **Significant Differences** | | |
| --- | --- | --- | --- | --- | --- | --- | --- |
| **Role** | **SEED Role** | **Sargasso - whale** | **Sargasso - farm** | **whale - farm** | **Sargasso cf whale** | **Sargasso cf. farm** | **Whale cf. farm** |
| ABC transporters, prokaryotic | ABC transporter branched-chain amino acid (TC 3.A.1.4.1) | -0.018 | -0.028 | 0.066 | Sargasso | farm | farm |
| ABC transporters, prokaryotic | ABC transporter dipeptide (TC 3.A.1.5.2) | -0.018 | -0.028 | 0.066 | whale | farm |  |
| ABC transporters, prokaryotic | ABC transporter ferric enterobactin (TC 3.A.1.14.2) | -0.018 | -0.028 | 0.066 | whale |  |  |
| ABC transporters, prokaryotic | ABC transporter ferrichrome (TC 3.A.1.14.3) | -0.018 | -0.028 | 0.066 | whale | farm |  |
| ABC transporters, prokaryotic | ABC transporter glutamate aspartate (TC 3.A.1.3.4) | -0.018 | -0.028 | 0.066 | Sargasso | Sargasso |  |
| ABC transporters, prokaryotic | ABC transporter glutamine (TC 3.A.1.3.2) | -0.018 | -0.028 | 0.066 | Sargasso | Sargasso |  |
| ABC transporters, prokaryotic | ABC transporter iron(III) dicitrate (TC 3.A.1.14.1) | -0.018 | -0.028 | 0.066 | whale |  | whale |
| ABC transporters, prokaryotic | ABC transporter L-proline glycine betaine (TC 3.A.1.12.1) | -0.018 | -0.028 | 0.066 | Sargasso | Sargasso |  |
| ABC transporters, prokaryotic | ABC transporter macrolide | -0.018 | -0.028 | 0.066 | whale | farm | farm |
| ABC transporters, prokaryotic | ABC transporter oligopeptide (TC 3.A.1.5.1) | -0.018 | -0.028 | 0.066 |  | farm |  |
| ABC transporters, prokaryotic | ABC transporter polyamine putrescine spermidine (TC 3.A.1.11.1) | -0.018 | -0.028 | 0.066 |  | Sargasso | whale |
| ABC transporters, prokaryotic | ABC transporter putrescine (TC 3.A.1.11.2) | -0.018 | -0.028 | 0.066 |  |  | whale |
| ABC transporters, prokaryotic | ABC transporter ribose (TC 3.A.1.2.1) | -0.018 | -0.028 | 0.066 |  | farm | farm |
| Arginine and proline metabolism | Arginine Biosynthesis | 0.049 | 0.059 | 0.076 |  |  | whale |
| Bacterial chemotaxis | Bacterial Chemotaxis | -0.525 | -0.28 | 0.245 | whale | farm |  |
| Benzoate degradation via hydroxylation | Benzoate catabolism | -0.067 | -0.114 | 0.096 | whale |  |  |
| Biotin metabolism | Biotin biosynthesis | -0.117 | -0.088 | 0.141 | whale |  |  |
| DNA polymerase | DNA Repair Base Excision | 0.07 | 0.109 | -0.021 |  | farm | farm |
| Fatty acid metabolism | fatty acid metabolism | -0.105 | 0.023 | -0.046 | whale |  | whale |
| Fatty acid biosynthesis (path 2) | fatty acid oxidation pathway | -0.18 | 0.047 | -0.242 | whale | farm | whale |
| Flagellar assembly | Flagellum | -0.355 | -0.079 | -0.108 | whale | farm |  |
| Folate biosynthesis | Folate Biosynthesis | -0.106 | -0.147 | 0.058 | Sargasso | Sargasso |  |
| Galactose metabolism | Galactose degradation | 0.009 | -0.097 | -0.045 | Sargasso |  | farm |
| Glutamate metabolism | Glutamate, aspartate and asparagine biosynthesis | 0.025 | 0.121 | -0.059 |  | Sargasso |  |
| Glutathione metabolism | Glutathione Redox Metabolism | -0.067 | 0.075 | -0.291 | whale |  |  |
| Histidine metabolism | Histidine Biosynthesis | -0.011 | 0.028 | 0.027 | Sargasso | Sargasso | whale |
| Histidine metabolism | Histidine Degradation | -0.011 | 0.028 | 0.027 | whale | farm |  |
| Inositol metabolism | Inositol catabolism | 0.191 | 0.045 | -0.146 | Sargasso |  | farm |
| Nitrogen metabolism | Nitrate and nitrite ammonification | -0.099 | 0.002 | -0.041 | whale | farm | farm |
| Biphenyl degradation | n-Phenylalkanoic acid degradation | -0.127 | -0.126 | -0.21 | whale |  | whale |
| Pyruvate metabolism | Pyruvate metabolism I: anaplerotic reactions, PEP | 0.018 | 0.089 | -0.015 |  |  | whale |
| Riboflavin metabolism | Riboflavin metabolism | -0.024 | 0.083 | 0.039 | whale |  | whale |
| RNA polymerase | RNA polymerase archaeal | 0.203 | 0.351 | -0.002 |  |  |  |
| RNA polymerase | RNA polymerase bacterial | 0.203 | 0.351 | -0.002 | Sargasso | Sargasso | whale |
| RNA polymerase | RNA polymerase chloroplast | 0.203 | 0.351 | -0.002 | Sargasso | Sargasso |  |
| RNA polymerase | RNA polymerase II | 0.203 | 0.351 | -0.002 | Sargasso | Sargasso |  |
| RNA polymerase | RNA polymerase III | 0.203 | 0.351 | -0.002 | Sargasso | Sargasso |  |
| Sulfur metabolism | Sulfur Metabolism | -0.015 | 0.131 | 0.013 | Sargasso | Sargasso |  |
| Thiamine metabolism | Thiamin biosynthesis | -0.005 | 0.035 | 0.002 | Sargasso | Sargasso |  |
| Ubiquinone biosynthesis | Ubiquinone Biosynthesis | 0.029 | -0.009 | -0.033 |  | farm |  |
| Valine, leucine and isoleucine degradation | Valine degradation | -0.103 | 0.018 | -0.013 | whale |  | whale |
